# A systematic review of phase II trials of thalidomide/dexamethasone combination therapy in patients with relapsed or refractory multiple myeloma

**DOI:** 10.1111/j.1600-0609.2008.01121.x

**Published:** 2008-10

**Authors:** Marie von Lilienfeld-Toal, Corinna Hahn-Ast, Kerstin Furkert, Florian Hoffmann, Ralph Naumann, Ralf Bargou, Gordon Cook, Axel Glasmacher

**Affiliations:** 1 Medizinische Klinik und Poliklinik III, Rheinische Friedrich Wilhelms UniversitätBonn, Germany; 2 BMTU, St James's University HospitalLeeds, UK; 3 Medizinische Klinik und Poliklinik I, Universitätsklinikum Carl Gustav CarusDresden, Germany; 4 Medizinische Klinik und Poliklinik II, Universitätsklinikum WürzburgGermany

**Keywords:** systematic review, multiple myeloma, relapsed/refractory, thalidomide, dexamethasone

## Abstract

Thalidomide monotherapy in relapsed/refractory multiple myeloma (MM) has a response rate of 30%. The combination of thalidomide with dexamethasone (Thal/Dex) is expected to improve responses, but it is unknown if the combination increases the rate of adverse events. Here, we conducted a systematic review of studies evaluating Thal/Dex in relapsed/refractory MM. Twelve studies were included, comprising 451 patients. The response rate (CR and PR) was 46% (95% CI 42–51%). Therapy-related toxicity was comparable to thalidomide monotherapy and included somnolence (26%, 95% CI 22–31%), constipation (37%, 95% CI 32–42%) and peripheral neuropathy (27%, 95% CI 23–32%). Only venous thromboembolism appeared to occur more often with Thal/Dex (5%, 95% CI 3–8%). Thus, using Thal/Dex results in an improved response rate in relapsed/refractory MM, with a toxicity rate comparable to thalidomide monotherapy.

Thalidomide as a single agent has been reported to be efficacious in relapsed or refractory multiple myeloma (MM) in 30% of patients (1, 2). To improve efficacy and outcome of thalidomide therapy, clinicians often add another agent to the monotherapy. The combination with dexamethasone (Thal/Dex) has been reported to increase the response rate (3–5), but although there have been several reports evaluating efficacy and toxicity of the combination therapy, the number of patients in the individual studies is often small and outcomes sometimes obscured by further addition of other agents like doxorubicine (6). Also, small sizes of individual studies make the extraction of additional information like survival outcome and rate and severity of adverse events unreliable. On the other hand, the toxicity rate is of particular interest as some authors report a higher rate of adverse events when thalidomide is combined with dexamethasone (7) and it would be important to know if this could possibly outweigh the benefit of the combination.

We have conducted a review of published studies in a systematic fashion in order to give an overview of the existing studies and to determine the pooled response rate of Thal/Dex. We also extracted information on survival and the toxicity profile, which might aid clinicians in their application of Thal/Dex in the setting of relapsed/refractory MM.

## Methods

### Search strategy

MEDLINE (PubMed version) was searched on 15 August 2007 with the combination of the following terms: thalidomid* AND (myelom* OR plasmocytom*) AND dexamethason*. Reference lists of all identified studies and related reviews were screened. The published abstracts of the annual meetings of the American Society of Hematology, the European Haematology Association, the American Society of Clinical Oncology, the German and Austrian Society of Haematology and Oncology and the British Society for Haematology were screened from 1998 to 2006.

### Inclusion/exclusion criteria

All trials published in the English language were included that evaluated combination therapy with thalidomide and dexamethasone in patients with relapsed or refractory MM and had a clear definition of complete (CR) and partial remission (PR). Trials were excluded if they reported only on patients with plasma cell leukaemia, solitary or extramedullary plasmocytoma.

### Extraction process

A validated structured form was used for an independent extraction by at least two reviewers for each trial. Disagreements were resolved by consensus with another reviewer. Reviewers were not blinded to authors or journals.

### Statistical analysis

Descriptive statistics were used and, for the main outcomes, medians and interquartile range (IQR) or 95% confidence intervals (95% CI) are reported. Data were analysed with the statistical package for the social sciences for Windows Software (Release 11.0.1; SPSS Inc., Munich, Germany). 95% CI were calculated with confidence interval analysis (version 2.1.1, T. Bryant, Southampton, UK). Correlation coefficients were calculated according to Spearman.

## Results

Forty-three trials were identified of which 18 did not allow extraction of data. Twenty-five studies were eligible, but 13 of them proved to be duplicate or secondary reports. Therefore, 12 studies entered the final analysis (3–5, 8–16) including altogether 451 patients.

Most studies were unicentric phase II studies and included a median of 25 patients each. None of them was a randomised controlled trial. Three studies, including 230 patients, were conducted in a multicentric setting. Publication date was between 2000 and 2007. Characteristics of included studies are illustrated in Table 1. No study reported the cumulative dose of thalidomide and only two studies reported the median dose (300 and 400 mg/d respectively), but the start and the target doses were reported by most studies [median starting dose 200 mg/d (IQR 100–200 mg/d), median target dose 350 mg/d (IQR 200–550 mg/d); Table 1]. Table 1 also depicts dose intensity of dexamethasone in the first month (median 180 mg; IQR 160–290 mg), which was applied in different schedules: 40 mg/d for days 1–4 every month in four studies (181 patients), 40 mg/d or 20 mg/m^2^/d for days 1–4 every 3 wk in three studies (66 patients), 12 mg/d days 1–4 every 3 wk in one study (12 patients) and 20 mg/m^2^ days 1–5 every 15 days (47 patients). In two studies with 79 patients, the dexamethasone dose (40 mg/d days 1–4 or 20 mg/m^2^/d) was repeated weekly or biweekly in the beginning of the treatment and then reduced to a monthly schedule. One study (*n* = 66) applied 4 mg/d dexamethasone in the first month with a quick tapering of 1 mg/d/wk to a maintenance dose of 1 mg/d.

**Table 1:** Description of included studies

	No. studies	No. patients (total 451)	Response rate CR + PR, %(95% CI)
Size of study (*n* patients)			
5–25	5	82	50 (39–61)
26–46	4	136	48 (40–56)
47–120	3	233	44 (38–51)
Thalidomide starting dose			
100	5	250	40 (35–47)
200	5	165	52 (45–60)
400	1	6	83 (44–97)
Thalidomide target dose			
100	1	120	52 (43–60)
200	4	156	42 (35–50)
300/400	4	84	64 (54–74)
600/800	3	91	40 (30–50)
Dexamethasone cumulative dose in first month			
<200 mg	6	259	45 (39–51)
200–320 mg	4	101	47 (37–56)
>400 mg	2	91	51 (41–61)

Only 11 studies stated a starting dose.

The median age of the study populations was 63 yr (IQR 62–65). Six studies provided information as to whether patients had relapsed or refractory disease. Here, most patients (*n* = 185 of a total of 239) had relapsed disease after prior therapy including high-dose therapy in at least 33 patients (of five studies that reported it). Fifty-four patients suffered from primary refractory disease.

**Figure 1:**
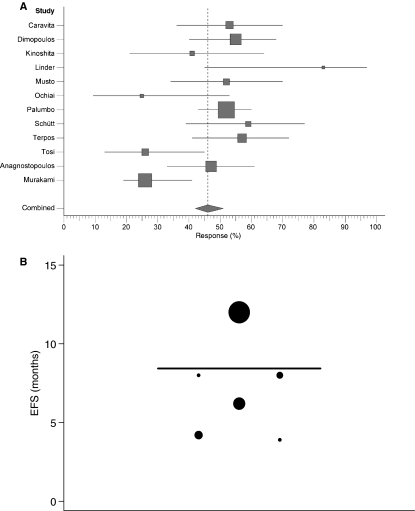
(A) Response rates of all included studies. Each dot indicates the response rate in one study, the size of the dot corresponds to the size of the study and the horizontal line depicts the 95% CI. The vertical line depicts the overall median and the diamond represents the 95% CI of the combined response rate. (B) Event-free survival of the studies that reported it (*n* = 6). Each dot indicates the median event-free survival in one study, the size of the dot corresponds to the size of the study. The line depicts the overall median.

Response was reported according to the European Group for Blood and Marrow Transplantation (EBMT) criteria in two studies, one study used the Southwest Oncology Group (SWOG) criteria. Most studies merely reported reductions of paraprotein, defining CR by disappearance of paraprotein as determined by immunofixation accompanied by <5% plasma cells in the bone marrow and PR as a reduction of the paraprotein level by 50% or 75%. Consequently, we know of 17 (4%) patients with a CR. Altogether, 209 patients responded with CR/PR, giving a median remission rate of 46% (95% CI 42–51%, Fig. 1A). In contrast, 39 patients from nine studies with 260 patients were progressive under treatment (15%, 95% CI 11–20%) and 44 patients from eight studies with 222 patients achieved stable disease (20%, 95% CI 15–26%). An analysis of the influence of country, multi- vs. unicentric design and study size revealed no significant difference of response rates. However, there appears to be a trend towards a better response with a higher starting dose of thalidomide (Table 1) and we found a positive correlation between the starting dose and the response rate (*r* = 0.7, *P* = 0.016, Spearman correlation), but not for the target dose (*P* = 0.7). In contrast, there was no significant effect of dose intensity of dexamethasone on the response rate (*P* = 0.3).

Four studies reported a median time to response: 1.3 months (44 patients), 2 months in two studies (77 patients) and 4 months (120 patients) respectively. Seven studies reported event-free survival (EFS): the 1-yr EFS was 28% in one study, six studies reported a median EFS with a weighted median value of 8 months (minimum 3.9, maximum 12 months, Fig. 1B). Overall survival (OS) was reported by seven studies: five studies reported a median OS with a weighted median value of 27 months (minimum 13, maximum 38 months); two studies reported a 1-yr OS of 63% and 83%.

**Figure 2:**
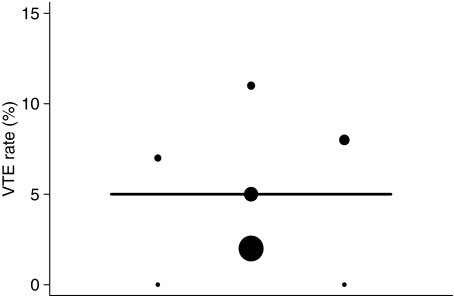
Rate of venous thromboembolic events (VTE) in those studies which reported it (*n* = 7). Each dot indicates the rate of VTE in one study, the size of the dot corresponds to the size of the study. The line depicts the overall rate.

Four hundred and thirty-two patients were evaluable for any reports of adverse events. Forty patients (13%, from eight studies with a total of 297 patients) discontinued treatment because of toxicity (Table 2). The most common adverse events were constipation, neuropathy and somnolence. The rate of thrombo-embolic events (VTE) was 5% (15 of 308 patients, 95% CI 3–8%, Fig. 2), which is twice as high as the rate of VTE with thalidomide monotherapy. We could not identify any relationship between the dose of either thalidomide or dexamethasone and the rate of VTE. Most studies did not report any infectious episodes (3, 4, 8–12, 15). One study reported one episode of skin infection (5), one reported an episode of fatal febrile neutropenia (16) and two reported several episodes of infection [one case of pneumonia, two of sepsis and one of herpes encephalitis in (13) and 10 respiratory infections, three skin infections and two urinary tract infection in (14)]. Numbers are too small to draw any conclusion with regard to an association between the dexamethasone dose and the rate of infection.

**Table 2:** Toxicity of the combination therapy with thalidomide and dexamethasone

	*n*/*N*	%	95% CI
Constipation	140/379	37	32–42
Thromboembolic event	15/308	5	3–8
Somnolence	93/352	26	22–31
Neuropathy	101/373	27	23–32
Depression	27/265	10	7–14
Discontinuation because of adverse event	40/297	13	10–18

*n*, events; *N*, total number of patients for which the incidence of this adverse effect was reported.

## Discussion

This systematic review confirms the improved response rate of the combination therapy of thalidomide and dexamethasone in refractory/relapsed multiple myeloma. The pooled data of 12 studies reveal a median response rate of 46% (95% CI 42–51%), which is a considerable improvement when compared with thalidomide monotherapy, where the median response rate lies at around 30% (1, 2).

Generally, reviews like ours suffer from inconsistencies amongst studies in reporting adverse events. Here, information about toxicity was available on more than 60% of the whole study population (Table 2), which renders most observations fairly reliable. Still, a potential reporting bias has to be kept in mind when interpreting the results. Despite the increase in clinical efficacy, the toxicity profile of the combination is very comparable to monotherapy. The rate of discontinuation was similar to the rate in monotherapy: here as well as in the previous review, a discontinuation rate of 13% was found. In contrast, constipation and somnolence, which have been reported to occur in 54% and 56% with thalidomide alone, occurred considerably less frequently (37% and 26% respectively). We suspect this to be caused by a lower dose of thalidomide in the combination therapy: whereas the median target dose in the monotherapy studies was 800 mg/d (1), the median target dose in this review was 350 mg/d. The only adverse event which occurred more often was the rate of VTE, which was 5% when combining thalidomide with dexamethasone [vs. 2–2.7% (1, 2) in monotherapy]. This is nonetheless a low figure, particularly when compared with the incidence of VTE in newly diagnosed MM treated with thalidomide combinations (14–26%) (17). We hypothesize that this might be in part caused by a lower tumour burden at relapse causing less of a procoagulant state. Also, the surprisingly low figure might be the result of a more efficient anticoagulation, as most studies were conducted after 2000, when awareness of the risk of VTE started to increase. In contrast, studies investigating thalidomide alone were mostly conducted before 2000 and no anticoagulation was used. However, most studies of our review did not report on any anticoagulation and therefore we can only speculate that the low rate of VTE might be the result of effective prophylaxis.

Our review shows a higher response rate in those patients who received a higher dose of thalidomide, supporting similar findings of studies with thalidomide monotherapy in relapsed/refractory myeloma (18). In contrast, we could not detect an influence of dexamethasone dose intensity on response rates or toxicity. Currently, there is ongoing discussion regarding the dose of dexamethasone in an attempt to optimise efficacy whilst minimising side effects. The studies reviewed here were heterogenous with regard to their dexamethasone dosing schedule, but when analysing the cumulative dose of dexamethasone in the first month we could not find a significant influence of dose on response rate or toxicity. Of note, half of the studies reviewed here had a dexamethasone dose intensity similar to the lower-dose arm in the ECOG study (19) evaluating the thalidomide derivative lenalidomide combined with high-dose (480 mg in the first month) vs. low-dose (160 mg in the first month) dexamethasone in newly diagnosed multiple myeloma. Here, patients receiving low-dose dexamethasone had a significant survival benefit. Our review, while not a randomised study, offers confirmatory evidence that lower doses of dexamethasone are also efficient in the relapsed/refractory setting. It is of interest that the rate of VTE was 6.1% in the ECOG low-dose arm, comparing well with the 5% in our review of thalidomide/dexamethasone.

In conclusion, the combination thalidomide/dexamethasone leads to an increased response rate compared with thalidomide monotherapy but not to a significant increase in toxicity, suggesting a synergy between these two agents which allows for more tolerable doses of both substances.
